# Non-Editorial Note

**Published:** 1947

**Authors:** 


### Non-Editorial Note

**Twenty-first Birthday.**

IN the nature of things it must be, if not unprecedented, at least very rare for one man to occupy the editorial chair of a medical journal for twenty-one years. Yet we are reminded that Professor J. A. Nixon was elected to that honourable, if onerous position, in the spring of 1926. This was immediately after we signalized recovery from the ravages of war in a larger page, larger type, and better paper: fit preparation for the development which ensued. Few indeed are the opportunities for a provincial medical paper to enjoy such illuminating and experienced guidance. Three events of particular importance has our *Journal* recorded during the period: the centenary of the Bristol Medical School, the bi-centenary of the Royal Infirmary, and the Jubilee of the *Journal* itself. Each of these received special treatment largely from the Editors' hands, the last being the occasion for the fifty-year Index.

Like his predecessor, the Editor has had the heavy task of guiding the *Journal* through the vicissitudes of a world war. His was probably the heavier task—the bombing of Bristol, severe rationing of paper and (not least), the complete cessation of meetings by the Society made it, at times, almost insuperable.

It may be of some interest to compare how the *Journal* fared under the stresses of two wars.

| Year | Issues | Pages | Issues: Total | Issues: Average per year | Pages: Total | Pages: Average per issue | Pages: Average per year |
|---|---|---|---|---|---|---|---|
| 1915 | 3 | 234 | | | | | |
| 1916 | 2 | 205 | | | | | |
| 1917 | 3 | 141 | 11 | 2+ | 766 | 69 | 153 |
| 1918, 1919 | 3 | 186 | | | | | |
| 1920 | 4 | 245 | | | | | |
| 1921 | 3 | 175 | 9 | 3 | 538 | 60 | 179 |
| 1922 | 2 | 118 | | | | | |
| 1940 | 2 | 142 | | | | | |
| 1941 | 2 | 104 | | | | | |
| 1942 | 2 | 84 | | | | | |
| 1943 | 1 | 50 | 9 | 1.5 | 476 | 53 | 79 |
| 1944 | 1 | 46 | | | | | |
| 1945 | 1 | 50 | | | | | |

Observe that four years passed after the first world war before the *Journal* really recovered from its effects : in 1923 the present Editor's Presidential Address heralded the return to regular quarterly issues. But the 1919 issue contained an announcement that the subscription would be raised for 1920, followed shortly by the rise of subscription for membership of the Society to one-and-a-half guineas—part of " the price of victory." This time the storm has been more severe while it lasted, and the increase in cost of production proportionately greater, but we trust the return to quarterly issues will not again prove premature.

